# Subcorneal Pustular Dermatosis in Paediatrics: A Case Report and Review of the Literature

**DOI:** 10.7759/cureus.20221

**Published:** 2021-12-06

**Authors:** Mais A Alhafi, Mohamed I Janahi, Zainab N Almossalli

**Affiliations:** 1 Dermatology, Salmaniya Medical Complex, Manama, BHR; 2 Orthopedic Surgery, Salmaniya Medical Complex, Manama, BHR

**Keywords:** dapsone, dermatology, paediatrics, sneddon-wilkinson disease, subcorneal pustular dermatosis

## Abstract

Subcorneal pustular dermatosis (SPD) is a rare chronic condition rarely seen in adolescence and childhood. The exact etiology of the disease remains unknown. In this paper, we report the case of a 14-year-old girl who came with a history of itchy skin lesions confined to the upper and lower extremities, thighs and pubic area for two months. Physical examination showed well-demarcated annular brownish plaques, ranging in size from 5cm to 7cm, in addition to a scaly and elevated border with few pustules noted over the upper and lower extremities, thighs and pubic area. Some lesions also showed central clearing. New annular vesicular lesions were also noted on the lower extremity and inner thigh. She was diagnosed with SPD based on the characteristic clinical and histological features. The patient was treated with Dapsone and showed good clinical response.

## Introduction

Subcorneal pustular dermatosis (SPD) or Sneddon-Wilkinson disease, is a rare, chronic yet benign inflammatory neutrophilic dermatosis (ND), which was first described by Lan Sneddon and Darell Wilkinson in 1956 [1]. Its specific etiology and pathophysiology remain largely unknown. The condition is four times more common in women than in men [2,3]. Although it typically presents in middle-aged women, there have been a few cases reported in children [4,5]. SPD has been associated with a wide spectrum of systemic disorders, including other neutrophilic dermatosis, hematologic disorders, connective tissue diseases, and neoplasms [6]. To the authors’ knowledge, a case of SPD without any medical background has been reported only once in the literature [7]. We report a case of a child diagnosed with SPD without any systemic illness.

## Case presentation

A 14-year-old female presented with a two-month history of itchy skin lesions confined to the upper and lower extremities, thighs and pubic area. Her system review was negative for any other complaints or associated symptoms.

**Figure 1:**
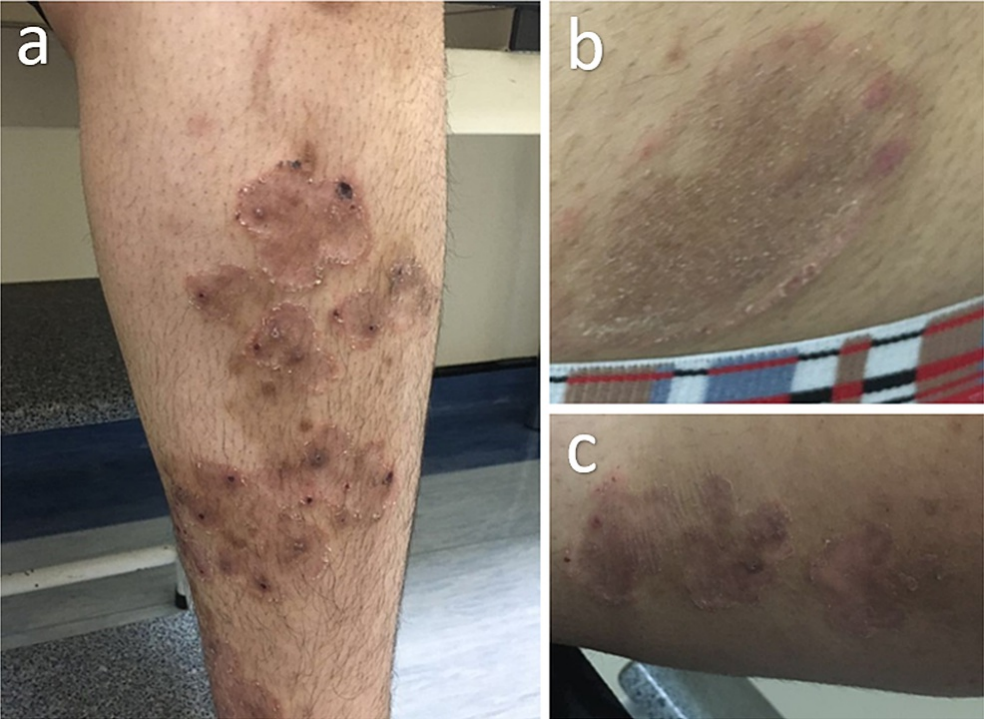
Pustular eruption in (a) lower leg, (b) pubic area, and (c) thigh at the initial presentation.

Physical examination revealed well-demarcated annular brownish plaques, ranging in size from 5cm to 7cm. Borders were scaly and elevated with few pustules noted over the upper and lower extremities, thighs and pubic area (Figure 1). In addition, some lesions showed central clearing. There was no hair, nail, mucosal or genital lesions.

In view of the previous findings, the patient diagnosis was Tinea corporis and she was started on terbinafine 250 mg tab plus topical miconazole cream twice daily for two weeks. She came back after two weeks with more lesions noted on the lower extremity and inner thigh despite her compliance with the treatment given.

**Figure 2:**
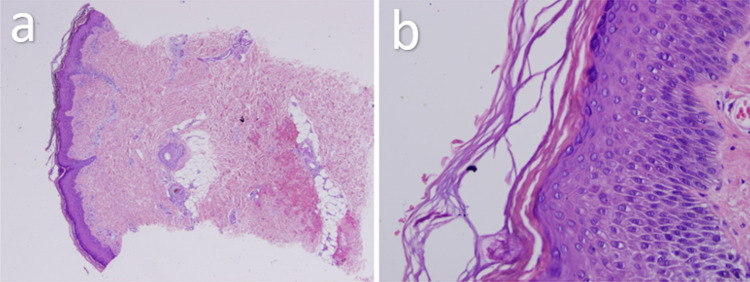
(a) Subcorneal pustular collections with epidermal spongiosis. (b) Magnified view.

A skin biopsy was taken from the left and right lower limbs. Histopathology demonstrated pustules located immediately below the stratum corneum and contained mainly polymorphonuclear leucocytes with a few eosinophils, acantholytic cells in the cavity, and spongiosis in the epidermis (Figure 2).

Periodic acid-Schiff (PAS) stain was negative for fungal infection. A complete blood count, erythrocyte sedimentation rate (ESR) activity, G6PD activity and liver function test showed normal results.

In view of the histopathological and clinical findings, the diagnosis was subcorneal pustular dermatosis (Sneddon-Wilkinson disease). The patient was started on dapsone 50mg once daily for two weeks then the dose was increased to 100mg once daily with good response.

## Discussion

Sneddon-Wilkinson or subcorneal pustular dermatosis (SPD) is a rare skin disorder, which typically presents in middle-aged women and is rarely seen in children [8]. Sarkany reported the first case of pediatric SPD in a 10-year-old patient. Since then, a few more cases of pediatric SPD were reported and it was found that the clinical condition is similar both in children and adults [9].

SPD is categorized as a neutrophilic dermatosis and characterized by pustules that appear in crops over months or years. It presents with chronic, recurrent vesiculopustular eruptions and might be a cause of sterile pustular eruptions in a child [1]. The lesions combine into circinate, annular or serpiginous patterns. SPD prefers the trunk and intertriginous areas, such as the groin, axillae, and submammary regions [8]. It has no estimate of prevalence, due to potential misdiagnosis of the disorder and its rare incidence [10].

The etiology of the disease is unknown and not fully understood to date, one of the theories considers the condition as an abnormal response of inflammation cells (neutrophils) to chemotactic factors (TNF-α, C5a, interleukin-8). The obvious response to anti-TNF drugs supports this theory [11]. In addition, there are well-documented SPD cases associated with benign monoclonal IgA gammopathy and pyoderma gangrenosum [12]. There are also reports that affiliate SPD with systematic conditions like IgA myeloma [8], SAPHO (synovitis, acne, pustulosis, hyperostosis, osteitis) syndrome [13], Crohn's disease [14], Sjogren's syndrome [15], rheumatoid arthritis [16], and thyroidal diseases [17].

Histopathologically, SPD hallmark is a subcorneal pustule filled with polymorphonuclear leukocytes. Otherwise, the underlying epidermis is generally spared, showing minimal spongiosis or acantholysis. SPD’s main clinical features are very particular and unique to make the diagnosis; the primary lesions compose of waves of isolated or grouped flaccid pustules measuring several millimeters with underlying potentially erythematous skin [18]. The histopathological examination of our case demonstrated focal hyperkeratosis, parakeratosis and subcorneal pustules. Not to mention, focal hypergranulosis, spongiosis, basal cell degeneration were noted and PAS stain was negative for fungal infection. A complete blood count, ESR activity, G6PD activity and liver function test showed normal results. Despite the association of SPD with various systemic disorders, such as immunoglobinopathies and lymphoproliferative disorders, like IgA multiple myeloma, our patient did not have any systemic diseases.

The differential diagnosis for SPD is quite broad and encompasses many variables. The primary cutaneous disorders to be considered include pustular psoriasis, IgA pemphigus and acute generalized exanthematous pustulosis (AGEP). Other localized forms include acute generalized exanthematous pustulosis, impetigo, dermatophytosis and dermatitis herpetiformis. Contrary to pustular psoriasis, scalp and nails are usually not affected in SPD, and the formation of micro abscess does not occur. A dermatophyte infection can be easily rejected using a direct microscopic examination of fungal elements. Impetigo differential diagnosis may be difficult and possible bacterial contamination might be demonstrated by Gram stain. SPD is distinguished from dermatitis herpetiformis by IgA deposition in the dermal papillae. Generally, physical examination, medical history, and histopathology can greatly narrow the differential diagnosis. Immunofluorescence staining can also be used to differentiate SPD from diverse immunobullous disorders [19].

Subcorneal pustular dermatosis has no cure. However, treatment is available to palliate it and it centers on the anti-neutrophilic sulfone, dapsone. Dapsone is the treatment of choice and in our case, it showed a good response [20]. Other treatments include acitretin, psoralen + ultraviolet light A (PUVA), narrowband ultraviolet B (UVB) [21], broadband UVB [22], colchicine [23], etretinate [24], cyclosporine, prednisone [25], and infliximab [26]. Potent topical corticosteroids may also be used as a treatment alone [27] or in combination with dapsone. Sulfapyridine and sulfamethoxypyridazine can also be used. However, they are not as effective [23]. To our knowledge, this is the first case report about pediatric subcorneal pustular dermatosis done in Bahrain.

## Conclusions

Although Sneddon-Wilkinson disease is extremely rare in children, it should be considered while investigating for the differentials. Proper investigations are crucial and should be done at first presentation in order to prevent poor outcome. Referral to other specialties must be initiated if any underlying rheumatologic or hematologic conditions have been identified. No curable treatment has been discovered yet and the management is purely palliative. Dapsone remains the first line of management and it should be started as soon as the diagnosis is made.
